# Ferric Carboxymaltose (FCM)–Associated Hypophosphatemia (HPP): A Systematic Review

**DOI:** 10.1002/ajh.27598

**Published:** 2025-02-11

**Authors:** Joseph Magagnoli, Kevin Knopf, William J. Hrushesky, Kenneth R. Carson, Charles L. Bennett

**Affiliations:** ^1^ The Southern Network on Adverse Reactions (SONAR), Clinical Pharmaceutical Outcomes Sciences Department University of South Carolina College of Pharmacy Columbia South Carolina USA; ^2^ The Division of Hematology/Oncology of the Department of Medicine Northwestern University Feinberg School of Medicine and the Robert H Lurie Comprehensive Cancer Center of Northwestern University Feinberg School of Medicine Chicago Illinois USA

**Keywords:** fatigue, ferric carboxymaltose (FCM), fibroblast growth factor 23 (FGF23), fracture, hypophosphatemia (HPP), intravenous (IV) iron, iron deficiency anemia (IDA), osteomalacia

## Abstract

**Background:**

Since 2015, ferric carboxymaltose (FCM), an intravenous (IV) iron formulation used for treating iron deficiency anemia (IDA), has been associated with an increasing number of reported hypophosphatemia (HPP) cases. Information on HPP clinical manifestations and incidence has not been reviewed.

**Methods:**

We reviewed HPP‐associated adverse events reported to the FDA, case reports, case series, observational databases, clinical trials, meta‐analyses, and FDA‐approved labels. Our analysis found that FCM‐associated HPP is a clinically important adverse drug reaction (ADR). The most common clinical manifestations are general weakness, fatigue, bone pain, muscle pain, osteomalacia, and fractures. Information on rates of FCM‐associated HPP was from a review of clinical trials, observational databases, systematic reviews, and meta‐analyses.

**Results:**

Clinical trials comparing FCM with other IV iron preparations identified FCM‐associated HPP rates between 50% and 92% versus 2% and 8% with other IV iron formulations. Meta‐analyses and systematic reviews confirmed these numbers. FDA‐approved FCM labels do not include details of available ADR information in case reports, case series, observational databases, randomized trials, and meta‐analyses.

**Conclusion:**

We conclude that although the FDA‐approved FCM Prescribing Label was updated in 2023, more robust recommendations on FCM‐associated HPP are needed to prevent negative outcomes including osteomalacia and fractures. For patient safety, FCM label should advise monitoring serum phosphate levels prior to initiating first doses and before subsequent doses for all patients. Given differences between the FDA‐approved FCM label and data reviewed herein, clinicians must be educated about FCM‐associated HPP, difficulties treating HPP cases, and should consider administering other IV iron formulations that have lower rates of HPP.

## 1 Introduction

Ferric carboxymaltose (FCM) is an intravenous (IV) iron formulation indicated for the treatment of iron deficiency anemia (IDA) [1, 2, 3]. Since FDA approval in 2013, the FCM prescribing information has undergone several updates, at times bringing more attention to the risk of FCM‐associated hypophosphatemia (HPP) [1, 2, 3]. It has become increasingly evident that FCM carries a special safety risk of HPP in patients by interfering with the hormone that regulates urinary phosphate excretion, fibroblast growth factor 23 (FGF23) [4, 5, 6, 7, 8, 9].

HPP is an acute or chronic condition that occurs when patients have low levels of serum phosphate (< 2.0 mg/dL) after IV iron therapy [10, 11]. Typical short‐term signs and symptoms of HPP include muscle weakness and fatigue, which overlap with the most common symptoms of IDA, making it difficult to discern one from the other. Medical attention may be necessary for both acute and chronic HPPs, with potentially life‐threatening implications in severe cases (< 1.0 mg/dL). Chronic or long‐term consequences of HPP include bone pain, osteomalacia, and pseudofractures [12, 13, 14, 15, 16, 17]. In the current FDA‐approved FCM label, the incidence of HPP is reported as ≥ 4% in adults and 13% in pediatric patients [1]. However, published data inclusive of clinical trials, systematic reviews, and meta‐analyses reported the rate of HPP with FCM to be as high as 92.1% for some adults [18]. Data show that levels of intact FGF23 (iFGF23) increase more with an FCM infusion compared to other IV irons, potentially leading to a cascade of metabolic events including high FGF23, HPP, hypovitaminosis D, hypocalcemia, and secondary hyperparathyroidism [19].

**FIGURE 1:**
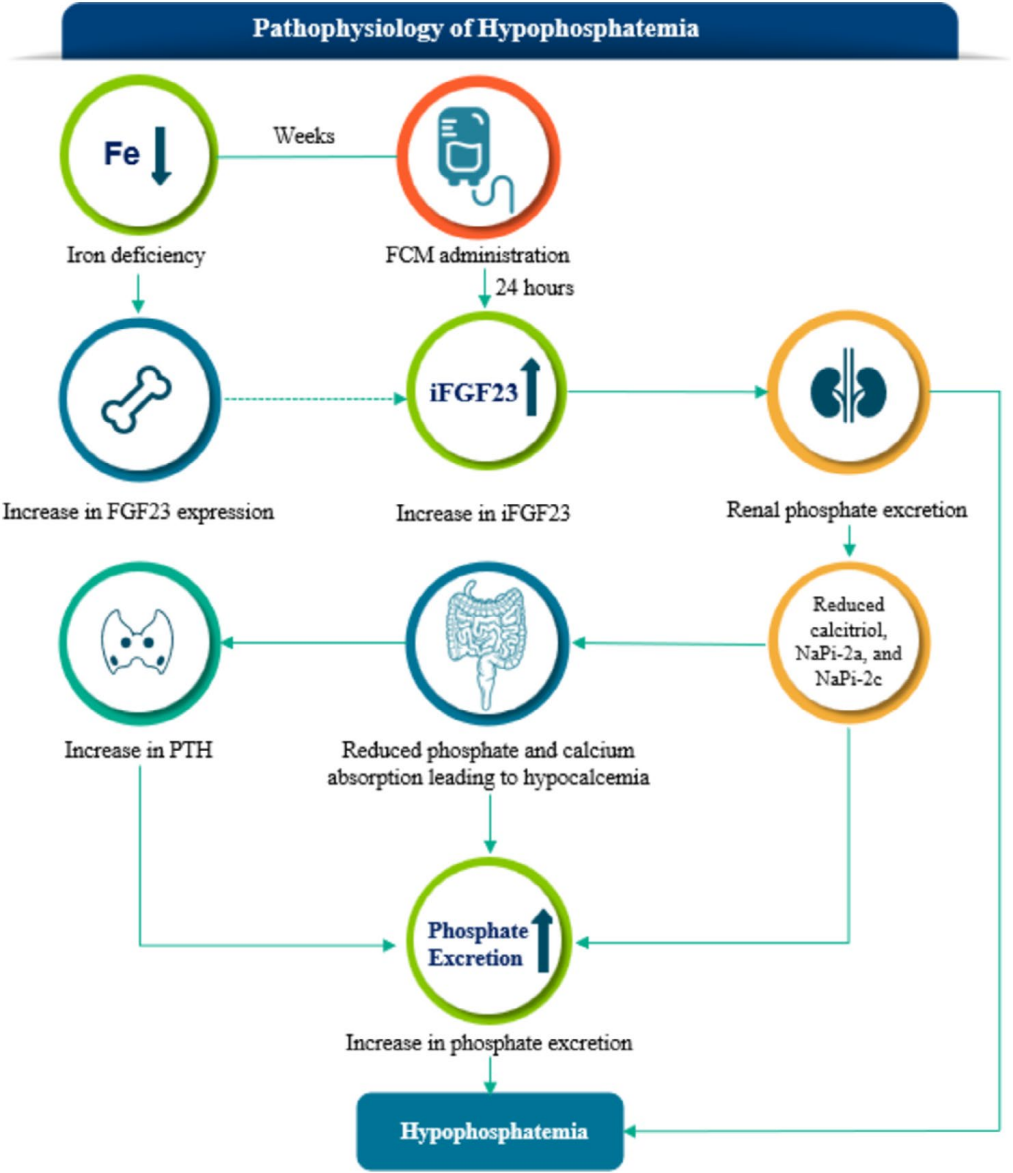
In most patients, FCM administration is followed by a sharp increase in intact FGF23 (iFGF23), which triggers a pathophysiological cascade of renal phosphate wasting, calcitriol deficiency, and secondary hyperparathyroidism frequently culminating in HPP even after iFGF23 levels have normalized. FGF23 = fibroblast growth factor 23; iFGF23 = intact fibroblast growth factor 23. Mechanism of FCM‐associated HPP adapted from Schaefer et al. [23]. [Color figure can be viewed at wileyonlinelibrary.com]

FGF23 is an osteocyte‐derived hormone that regulates phosphate and vitamin D homeostasis [19]. Though the exact mechanism of FCM‐associated HPP is unknown, a hypothesis is that in the clinical setting, FCM prevents the cleavage of iFGF23 to the C‐terminal FGF23 (cFGF23) and N‐terminal FGF23 (nFGF23), resulting in increased circulating iFGF23 levels, which in turn cause lower serum phosphate levels (Figure 1), similar to genetic diseases of primary FGF23 excess (autosomal dominant hypophosphatemic rickets) or tumor‐induced osteomalacia [20, 21, 22].

Increased levels of iFGF23 prevent phosphate reabsorption in the proximal tubule leading to an increase in urinary phosphate excretion [23]. Elevated iFGF23 also inhibits the activation of 25 (OH) vitamin D to calcitriol, which lowers calcium levels and increases parathyroid hormone (PTH) levels. This secondary hyperparathyroidism can lead to further phosphate excretion, potentially prolonging HPP after levels of iFGF23 have returned to normal (Figure 1) [23]. This effect on bone mineral markers has been associated with FCM use and is often termed 6‐H syndrome, inclusive of high FGF23, hyperphosphaturia, HPP, hypovitaminosis D, hypocalcemia, and secondary hyperparathyroidism [10, 23, 24, 25].

Evidence of the underlying mechanism responsible for the FCM‐associated HPP was first reported in 2013 [4]. A study in women with a history of heavy uterine bleeding was conducted, evaluating IDA and its association with iFGF23 and cFGF23 levels over 35 days; patients received equivalent doses of randomly assigned, IV elemental iron in the form of FCM or iron dextran. At baseline, iron deficiency was associated with markedly elevated cFGF23 (807.8 ± 123.9 relative units [RU]/mL) but normal iFGF23 (28.5 ± 1.1 pg/mL) levels. Within 24 h of iron administration, cFGF23 levels fell by approximately 80% in both groups; however, in contrast, iFGF23 transiently increased in the FCM group alone and was followed by a transient, asymptomatic reduction in serum phosphate. The study had three novel findings: (1) iron deficiency is associated with normal iFGF23 and elevated cFGF23 to levels rarely seen except in renal failure or hereditary rachitic diseases. Similar results in wild‐type mice suggest that iron deficiency stimulates iFGF23 transcription but does not result in HPP since iFGF23 is cleaved to cFGF23 within osteocytes by an unknown catabolic system [26]. (2) Rapid correction of iron deficiency with two different IV iron preparations reduced cFGF23 levels by approximately 80% within 24 h. (3) FCM, but not iron dextran, induced significant increases (3–6‐fold) in iFGF23 levels 24 h after the FCM infusion [4].

Clinical manifestations of FCM‐associated HPP are diverse. The most common clinical manifestations are general weakness, fatigue, bone pain, muscle pain, osteomalacia, and fractures [23]. Clinically, FCM‐associated HPP may be difficult to diagnose because the most common symptoms of fatigue and weakness are also the symptoms most frequently reported in the disease state FCM is used to treat, IDA [9, 10, 11, 12, 18]. Patients with IDA and worsening fatigue or no improvement in fatigue symptoms post FCM infusion warrant further evaluation of phosphate levels and potential continued monitoring for FCM‐associated HPP [18, 23].

Based on available data, using a recently developed ADR system called the Southern Network on Adverse Reactions (SONAR) method, our research group has focused on the discipline of hematology [26]. We have identified FCM‐associated HPP as a safety hematological issue that warrants increased awareness and clinical intervention. This systematic review is to report a systematic review on FCM‐associated HPP.

## 2 Methods

### 2.1 Search Design and Strategy

The review protocol was developed based on the SONAR method for evaluating important adverse drug reactions (ADRS) [26]. This systematic method for ADRS differs from systematic methods developed for systematic reviews on efficacy. The 10‐point method requires comprehensive reviews of ADR information contained in the following data sources: adverse event reports to the Food and Drug Administration's Adverse Event Reporting System (FAERS) database, case reports, case series, randomized clinical trial ADR descriptions, systematic reviews, and clinical reviews [10, 27, 28, 29, 30, 31, 32, 33, 34, 35, 36, 37].

### 2.2 Study Selection

Two authors (CLB, WJH) screened the eligibility of the available studies for inclusion as guided by the SONAR framework. Studies were included if they contained information on the HPP ADR. Full text articles were retrieved for all published articles. FAERS ADR reports were obtained from the publicly available FAERS database.

## 3 Results

### 3.1 Case Reports

The first case report of FCM‐associated HPP appeared internationally in 2010 [30], and since then, the number of cases has continued to increase [12]. Eleven case reports provided information on treatment‐emergent HPP caused by FCM. FCM‐caused acute HPP primarily presents with severe fatigue; FCM caused by repeated dosing presents with chronic HPP associated with osteomalacia and fractures.

In one such case, a 28‐year‐old woman with a history of abnormal uterine bleeding and IDA presented to the emergency room with fatigue, muscle weakness, and palpitations [32]. Two doses of 750‐mg FCM were administered 1 week apart, and approximately 8 weeks prior to presentation. She was hospitalized and aggressively treated with IV and oral phosphorus and titrated calcitriol, but serum phosphorus remained low; range 1–1.6 mg/dL. On day 7 after admission, her serum phosphate level decreased critically, as low as 0.6 mg/dL, and the patient developed respiratory failure. The dose of IV calcitriol was slowly titrated up to 3 mcg/day, and 2 weeks later, the patient was discharged with serum phosphate concentration at borderline low levels. This case report offers valuable clinical insights into patient‐related symptoms. These findings have been further elucidated in case series and clinical trial reports [4, 11, 20, 27].

### 3.2 Database Analyses

A retrospective analysis of electronic medical records of individuals receiving FCM or FDI was conducted between January 2010 and December 2019 at the University Hospital in Innsbruck, Austria [36]. The study included 289 cases (179 cases received FCM and 110 received FDI), and the median follow‐up time was 5.8 years. The incidence of HPP (serum phosphate ≤ 2.0 mg/dL) was higher in the FCM‐treated versus the FDI‐treated cases at week 2 after the administration and onwards. Both FCM and FDI were efficacious and produced a similar increase in iron parameters, but the fracture rate for FCM‐treated cases was twice that of FDI‐treated patients (1.30 vs. 0.67) despite a similar risk in the two groups the year prior to the IV iron treatment (control period). Additionally, an experimental study was conducted where iron‐deficient animals were treated with FCM, FDI, or low‐molecular‐weight iron dextran (LMWID) [38]. Bone marrow–free femora of FCM‐treated animals had significantly higher iron concentrations than the animals treated with the two other formulations, FDI or LMWID. Seven days post treatment, significantly more iron was localized in the trabecular bone surface after FCM than after FDI or LMWID. Of the three iron formulations, only FCM had a distinct phosphate‐binding, pH‐dependent charge and inhibited dentin‐matrix protein‐1 (DMP1) binding. DMP1 is a known FGF23 inhibitor and important for bone turnover. This shows that FCM treatments may affect bone turnover beyond causing HPP and intact FGF23 induction.

A study conducted by the University College London retrospectively evaluated FCM‐associated HPP in hospitalized individuals with IDA from 2016 to 2017 [31]. Data were collected for 162 cases who received 169 FCM courses. The incidence of the primary outcome (moderate/severe HPP [serum phosphate < 2.0 mg/dL]) post‐FCM infusion was 33.7%. Within this group, the rate of severe HPP (serum phosphate ≤ 1.0 mg/dL) was 8.8%. Moderate/severe HPP persisted in 35% of cases having serum phosphate of < 2.0 mg/dL when evaluated at the 61–90‐day timepoint. The overall study conclusions were that moderate/severe HPP is a frequent adverse drug reaction with FCM and that FCM‐associated moderate/severe HHP was persistent, often requiring treatment, and was associated with longer hospital stay.

### 3.3 Randomized Clinical Trials

Compelling evidence for formulation‐related differences in HPP rates among various IV iron formulations (FCM, FDI, ferumoxytol [FXM], iron dextran) comes from several head‐to‐head randomized clinical trials evaluating the incidence of moderate‐to‐severe HPP [4, 11, 20, 27, 33, 34]. In a 2013 head‐to‐head trial, FCM, in comparison to iron dextran, was linked to a higher increase in iFGF23 levels within 24 h post‐infusion, leading to HPP (serum phosphate < 2.0 mg/dL) in 40% of the FCM‐treated patients [4]. A predefined sub‐analysis of the FIRM trial, published in 2018, confirmed that FCM was associated with a significantly higher incidence of HPP (serum phosphate < 2.0 mg/dL) and severe HPP (serum phosphate < 1.3 mg/dL) when compared to FXM (50.8% vs. 0.9% and 10.0% vs. 0.0%, respectively [*p* < 0.001]) [27]. HPP persisted on day 35 in 29.1% of FCM‐treated cases. Subsequent recent evidence comes from the two PHOSPHATE‐IDA trials (effects of iron isomaltoside versus FCM on HPP in iron‐deficiency anemia trials), published in 2020 [20]. These two identically designed trials were powered to directly compare the incidence of HPP. Again, pooled data showed that FCM was associated with a significantly higher incidence of HPP (serum phosphate < 2.0 mg/dL) and severe HPP (serum phosphate < 1.0 mg/dL) when compared to FDI (74.4% vs. 8.0% and 11.3% vs. 0.0%, respectively [*p* < 0.001]) A sub‐analysis of the PHOSPHATE‐IDA data showed that in the FCM‐treated cases who developed incident HPP, the condition was persistent for up to 35 days in 57.3% of patients and persistent HPP was not observed in FDI‐treated patient [33]. Different rates of HPP for FCM versus FDI reported in the PHOSPHATE‐IDA trials are in accordance with ex‐US data, specifically in the double‐blind European study, the HOMe aFers 1 study (HPP after high‐dose iron repletion with FCM and ferric derisomaltose—the randomized controlled) (HPP: 75.0% for FCM vs. 7.7% for FDI; *p* = 0.001) [34]. The most recently published head‐to‐head trial (published 2022), called the PHOSPHATE‐IBD trial (hypophosphatemia following ferric derisomaltose and FCM in cases with IDA due to inflammatory bowel disease (IBD)), was a double‐blinded, randomized clinical trial comparing equal doses of two different IV iron formulations (FCM and ferric derisomaltose (FDI)) in IBD cases [11]. Despite comparably effective results for the treatment of IDA in terms of hematological parameters, FCM caused a significantly higher rate of HPP (51.0%, *n* = 25/49) than FDI (8.3%, *n* = 4/48) (*p* < 0.0001). Additionally, patient‐reported fatigue scores improved in both groups but more slowly and to a lesser extent with FCM than FDI. The slower improvement in fatigue seen in the FCM group was associated with a greater decrease in phosphate concentration. The lowest level of phosphate in all the studies was around day 14 after the FCM infusion [4, 11, 20, 27, 33, 34].

### 3.4 Systematic Reviews

A systematic review of HPP was conducted based on a PubMed search including English‐language articles published within 10 years of the search date, February 28, 2024 (guided by the Preferred Reporting Items for Systematic Reviews and Meta‐Analyses [PRISMA] guidelines) [10]. At the time of the literature search, US‐marketed IV iron formulations included low‐molecular‐weight iron dextran, ferric gluconate, FCM, FXM, and iron sucrose. Of 511 publications (using keywords of HPP, FCM, and IDA), only 40 publications met the final inclusion criteria of reporting both serum phosphate levels and rates of HPP. The 40 publications included 19 randomized control trials, 10 retrospective, observational, or post hoc studies, and 11 case reports. The results showed that the rates of HPP ranged from 0.0% to 92.1% for FCM, 0.0% to 40.0% for iron sucrose, 0.4% for FXM, and 0.0% for low‐molecular‐weight iron dextran. Randomized controlled studies described HPP as “asymptomatic” or did not report on other associated sequelae. The analysis found a lack of standard approaches and timelines for measuring phosphate levels and significant variability in reporting, definitions, and follow‐up of HPP. Until the true prevalence and clinical impact of IV iron‐induced HPP are more fully characterized and quantified within the literature, findings of this systemic review suggest that physicians and researchers actively consider the possibility of HPP when administering IV iron, particularly when administering formulations such as FCM, which has been associated with the highest rates of HPP in various clinical studies.

### 3.5 Meta‐Analyses

A systematic literature search for articles indexed in EMBASE, PubMed, and Web of Science in years 2005–2020 was reported [10]. Search terms included “FCM” OR “iron isomaltoside (IIM).” The term iron isomaltoside is often used interchangeably with ferric derisomaltose in many countries outside the US. Prospective clinical trials reporting outcomes on HPP rate, mean nadir serum phosphate levels, and/or change in mean serum phosphate from baseline were selected. HPP rate and severity were compared for studies on FDI vs. FCM after stratification for chronic kidney disease. Meta‐regression analysis was used to investigate risk factors for HPP. Across 42 clinical trials including both CKD and non‐CKD, FCM induced a significantly higher incidence of HPP versus FDI (47%, 95% CI 36%–58% vs. 4%, 95% CI 2%–5%), and significantly greater mean decreases in serum phosphate (1.24 vs. 0.19 mg/dL). HPP persisted at the end of the study periods (up to 3 months) in up to 45% of patients treated with FCM. Meta‐regression analysis identified low baseline serum ferritin and transferrin saturation and normal kidney function as significant predictors of HPP. The authors concluded that FCM is associated with a high risk of HPP, which does not resolve for at least 3 months in a large proportion of affected individuals. In addition to the formulation choice, more severe iron deficiency and normal kidney function independent were risk factors for HPP identified in the meta‐analysis.

### 3.6 FDA Adverse Event Reporting System (FAERS) Data Review

**FIGURE 2:**
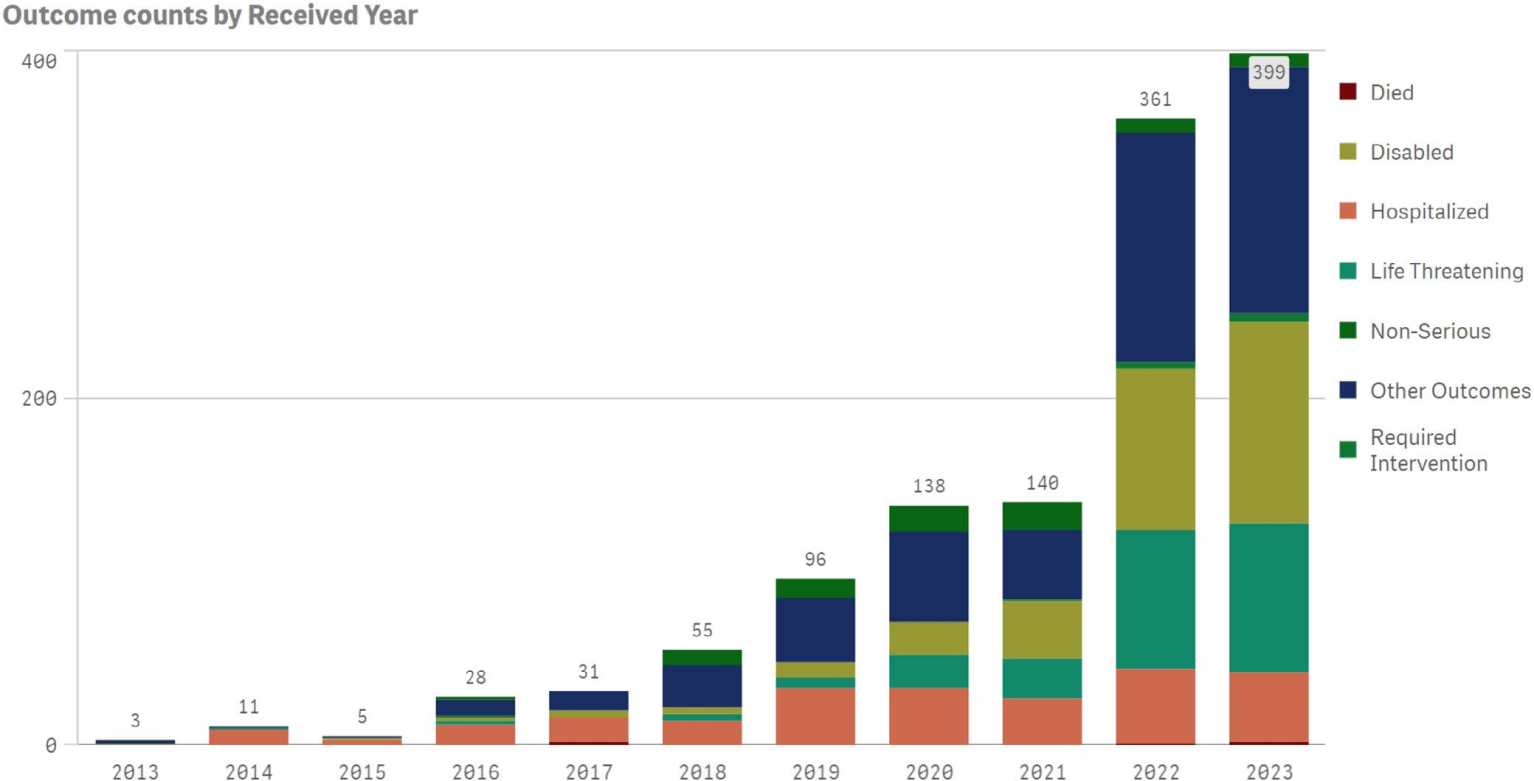
Case report of FCM‐associated HPP reported to FDA between 2014 and 2023. [Color figure can be viewed at wileyonlinelibrary.com]

The FDA provides public access to adverse event reports that have been submitted by pharmaceutical manufacturers, healthcare providers, attorneys, patients, and family members. SONAR reviewed FAERS data for FCM‐associated HPP and other serious adverse events [36]. Between 2014 and 2023, FAERS included 1270 reports related to FCM‐associated HPP (Figure 2). The number of reports of FCM‐associated serious (primarily associated with death, life‐threatening complications, or hospitalization) cases of HPP increased annually from 10 in 2014 to 244 by 2023 [37]. It is clear that FCM‐associated HPP is underreported to the FDA when compared to the high incidence reported in randomized clinical trials, systemic reviews, and meta‐analysis [4, 10, 18, 20, 27].

## 4 Discussion

IDA refractory to oral iron therapy is currently treated by IV iron preparations. FCM is a commonly used preparation that has been noted to cause high rates of HPP via its effect on the hormone FGF23, a clinically significant serious adverse drug reaction that has been increasingly recognized since FDA approval in 2013 [4, 5, 6, 7, 8, 9].

Our systematic review has important clinical implications. Based on the annual data provided by FAERS and SONAR estimates that generally only 1% of serious ADRs are reported to the FDA, as many as 39 300 FCM‐associated HPP cases could occur in the US annually [36, 39, 40]. However, fewer than 250 serious adverse drug reactions related to FCM were reported in 2023 signifying lack of awareness among US clinicians and patients [39]. FCM‐associated HPP can occur in any patient treated with FCM and can cause clinical sequalae (6H syndrome), which can impact patient quality of life and even lead to osteomalacia and fractures [12]. There is no standard of care for FCM‐associated HPP as it is refractory to both oral and IV phosphate [28, 29]. Increased awareness, monitoring, and selecting appropriate IV iron therapy are recommended to treating physicians to improve patient safety outcomes.

SONAR previously reported the significance of investigating patient impact of serious ADRs [26]. In SONAR interviews conducted with two patients, we gained insight into the clinical details of FCM‐associated HPP and associated cost of clinical care, including additional laboratory tests, emergency room visits, inpatient stays, and visits to primary care doctors, and specialist physicians [39]. These patients completed the SF‐36 questionnaire to assess health status at the time when phosphate levels were lowest. Both reported high levels of impact on general health, bone pain, low levels of energy/increased fatigue, and limitations to physical health, in general. They reported moderate‐to‐severe impact on social functioning and emotional well‐being.

The FDA‐approved FCM label states that clinicians should monitor serum phosphate levels in patients at risk for low serum phosphate who require a repeat course of FCM [1]. According to the FCM label, possible risk factors for HPP include a history of gastrointestinal disorders associated with malabsorption of fat‐soluble vitamins or phosphate, IBD, concurrent or prior use of medications that affect proximal renal tubular function, hyperparathyroidism, vitamin D deficiency, and malnutrition [1]. A drop in serum phosphate levels can occur as soon as 24 h after an FCM administration, and the lowest nadir is often seen around day 14 [11, 20, 27]. There are no evidence‐based recommendations on managing FCM‐associated HPP; therefore, guidance on treatment is based on experience and expert opinions [12, 18, 28]. In a 2021 narrative review, the authors suggest that the treatment of FCM‐associated HPP should be guided based on severity and symptomatology with no further FCM therapy [18]. Severe HPP is usually treated with oral or IV phosphate supplementation; however, it is important to note that phosphate supplementation may not be successful because the increased iFGF23 levels may continue to drive renal phosphate wasting and increase PTH, ultimately worsening HPP [28, 29]. Overall, normalizing FGF23 levels will help resolve HPP, but this can take several weeks or even months for resolution with FCM‐associated HPP. Primary prevention of HPP by using iron formulations other than FCM is preferred [12, 18, 28]. FCM should be used with caution in patients at risk for HPP, and serum phosphate should be monitored beginning before the first dose and with every dose thereafter in all patients receiving FCM.

FCM‐associated HPP is often overlooked by clinicians because the symptoms of HPP (most prominently fatigue and weakness) mimic symptoms of IDA. Our systematic review indicates that FCM‐associated HPP may occur after a single administration of FCM; therefore, serum phosphate levels should be measured prior to each dose of FCM in all individuals exposed to the drug. Second and subsequent infusions of FCM should be discontinued if HPP occurs after an earlier infusion. Additionally, FCM‐associated HPP is very difficult to treat and primary prevention of HPP by using iron formulations other than FCM is preferred [29]. All instances of FCM‐associated HPP in the US should be reported to the FDA's MedWatch program [40]. Information reviewed and recommended herein strongly supports the approach of our National Cancer Institute–sponsored pharmacovigilance center called SONAR [26]. The FAERS data highlight the increasing recognition of FCM‐associated HPP since 2017, but continued evaluation of HPP cases and reporting to FAERS are needed. The FCM label updates concerning HPP often go unnoticed even though the guidance is becoming more stringent [1, 2, 3]. There is even a gap that exists in the IV iron sections in the main treatment guidelines (NCCN, KIDGO) when it comes to addressing FCM‐associated HPP [41]. FCM‐associated HPP is not adequately addressed in these guidelines, and only limited guidance is provided regarding patients at risk and monitoring parameters for serum phosphate. Hopefully, this will change soon as emerging data recently presented show an increased fracture risk associated with FCM therapy [41].

## Author Contributions

All authors contributed to the project conception and design. Charles L. Bennett wrote the first draft. All co‐authors contributed to revising the first draft to generate the final draft. Drs. Bennett and Carson reviewed the statistics included in the manuscript. All authors read and approved the final initially submitted manuscript and the final revised manuscript.

## Ethics Statement

Because the manuscript included no identifiable human information, it was designated as Expedited Human Subjects review (University of South Carolina IRB ID: Pro00132017).

## Conflicts of Interest

The authors declare no conflicts of interest.

## Data Availability

The data that support the findings of this study are openly available in SONAR at https://www.SONAR.edu, reference number [26].
